# Associations between questionnaires on lifestyle and atherosclerotic cardiovascular disease in a Japanese general population: A cross-sectional study

**DOI:** 10.1371/journal.pone.0208135

**Published:** 2018-11-28

**Authors:** Hayato Tada, Masa-aki Kawashiri, Kenji Yasuda, Masakazu Yamagishi

**Affiliations:** 1 Department of Cardiovascular and Internal Medicine, Kanazawa University Graduate School of Medicine, Kanazawa, Japan; 2 Kanazawa Medical Association, Kanazawa, Japan; University of Tampere, FINLAND

## Abstract

**Objective:**

We aimed to investigate the association between questionnaires related to lifestyle habits and atherosclerotic cardiovascular diseases (ASCVD).

**Design:**

Cross-sectional observational study.

**Settings:**

Community-based medical checkups, called specific health checkups started in Japan since 2008. This checkup includes standard medical examinations as well as a specific questionnaire related to lifestyle habits.

**Participants:**

Overall, 47,842 subjects (males = 16,913, 35.4%) aged ≥40 years who underwent a Japanese specific health checkup in 2014 in Kanazawa city were included.

**Main outcome measures:**

Association between 12 lifestyle habits-related questionnaires and the presence of ASCVD, including coronary artery disease and stroke. The questionnaire included the following 12 questions on lifestyle habits: 1) weight gain (>10 kg/20 years), 2) exercise (>30 min, twice a week, >1 year), 3) daily walking or equivalent (>1 h), 4) walking faster (than others in the same generation), 5) body weight changes (>3 kg/year), 6) eating faster (than others in the same generation), 7) eating within 2 h before going to bed (more than three times a week), 8) having a snack after dinner (more than three times a week), 9) skipping breakfast (more than three times a week), 10) daily drinking (alcohol), 11) heavy drinking (more than 60 g ethanol/day), and 12) good sleeping.

**Results:**

Multivariable logistic regression analyses revealed that walking faster (odds ratio [OR] = 0.74, 95% confidence interval [CI] = 0.69–0.79, p < 0.0003), body weight changes (>3 kg/year, OR = 1.26, 95% CI = 1.16–1.37, p < 0.0003), eating faster (OR = 1.09, 95% CI = 1.03–1.15, p = 0.003), daily drinking (OR = 0.83, 95% CI = 0.76–0.89, p < 0.0003), and good sleeping (OR = 0.86, 95% CI = 0.79–0.93, p < 0.0003) were independently associated with ASCVD. Subjects with a high lifestyle habits risk score (number of bad habits: 7–12) had significantly higher odds for ASCVD than those with a low risk score (number of bad habits: 0–3, OR = 1.78, 95%CI = 1.62–1.95, p < 0.0003).

**Conclusion:**

Simple questionnaires related to lifestyle habits were associated with self-reported ASCVD.

## Introduction

The incidence of deaths because of atherosclerotic cardiovascular diseases (ASCVD) in Japan is still relatively rarer than that in Western countries, although changes in lifestyle habits may contribute to its increase. Owing to these conditions, community-based medical checkups, namely “specific health checkups,” have been started in Japan since 2008 based on the aging society and increase in lifestyle-related diseases. This checkup includes standard medical examinations, such as measurements of height, weight, waist circumference, blood pressure, and plasma metabolic markers (lipids and glucose) as well as a specific questionnaire related to lifestyle habits [1–3]. These questionnaires were selected based on previous nationwide surveys on diabetes and obesity or on reports from the subcommittee meeting of the Japanese Ministry of Health, Labour and Welfare without any validations so far. Healthy lifestyle factors have shown to be associated with a reduced risk of ASCVD in Western countries [4–9]; however, there is little data on this issue in a middle-aged Japanese population, whose “healthy lifestyles” could be different from those of Caucasians.

These questionnaires include several unique lifestyle habits that have not been assessed in previous studies, such as walking faster than people of the same generation and body weight changes (>3 kg/year) [10, 11]. Moreover, we hypothesized that a composite score that incorporates a wider range of lifestyle factors may show a stronger association with ASCVD. The aim of this study was to investigate the association between these questionnaires and ASCVD in specific health checkups in Japan. In addition, it is true that stroke as well as coronary artery disease share the risk factors, and it is important to assess both outcomes as ASCVD; however, it should also be interesting to see the difference between stroke and coronary artery disease (CAD). Thus, we also investigated the association between the questionnaires and each outcome.

## Materials and methods

### Study subjects

**Fig 1:**
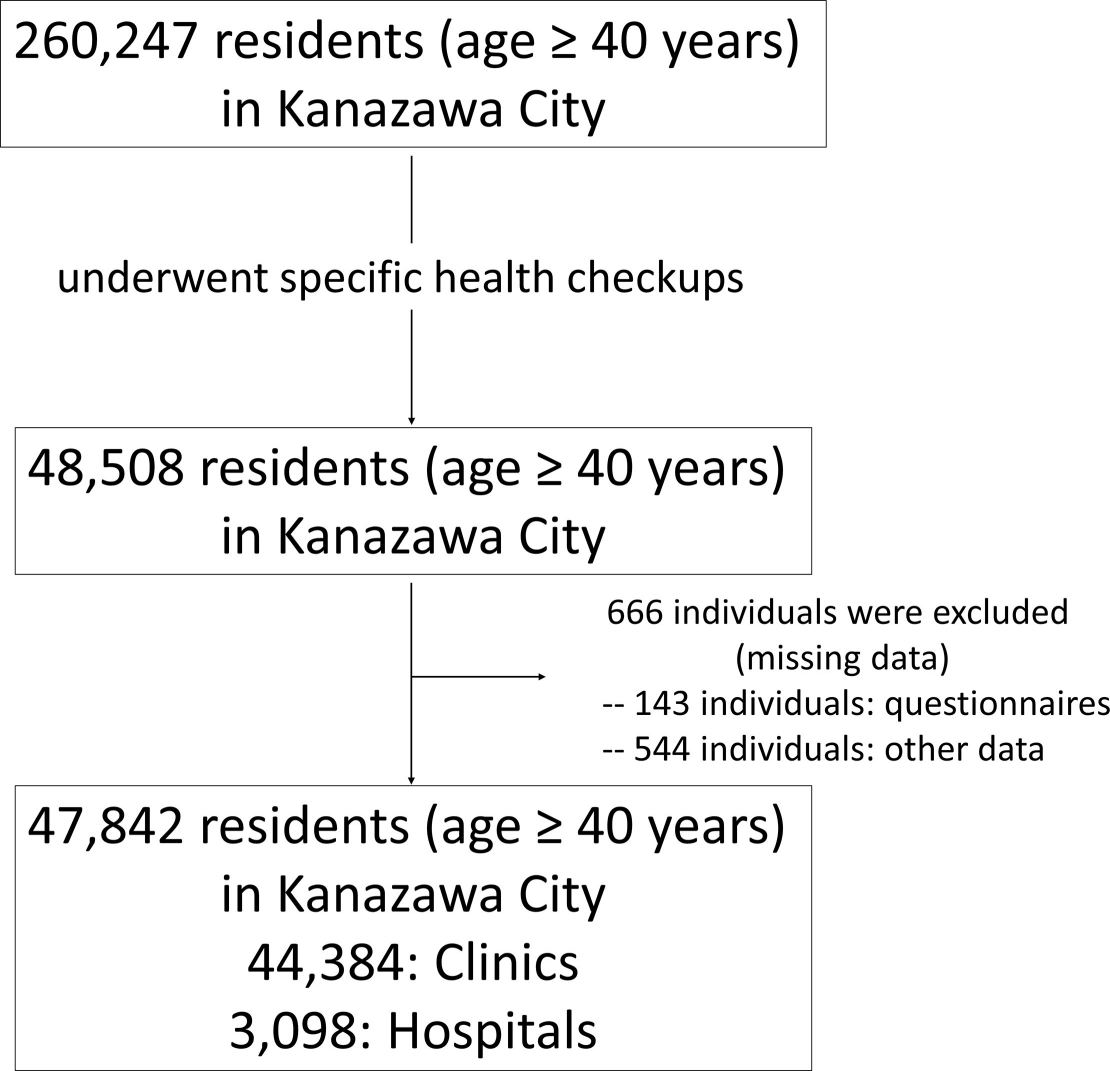
Flow diagram of the study. Among the 260,247 residents (age ≥ 40 years) in Kanazawa City, 48,508 individuals underwent the specific health checkups. Six hundred and sixty six individuals were exlcluded due to missing data. Finally, 47,842 individuals were included in this study.

Out of the 260,247 residents in Kanazawa City aged ≥40 years, 48,508 individuals underwent specific health checkups in 2014 in Kanazawa City (**Fig 1**). Eligibility for this health checkups includes 1) residents in Japan, 2) age ≥40, 3) national pension subscriber (non-regular worker). Overall, 47,842 subjects (males = 16,913, 35.4%) without any missing data were included in this study. Most of the subjects (44,384 among 47,842 individuals, 93%) visited general practitioners at clinics in Kanazawa city. All data were collected and anonymized by the Kanazawa Medical Association.

### Ethical considerations

This study was approved by the Ethics Committee of Kanazawa Medical Association and Kanazawa University and conducted in accordance with the Declaration of Helsinki (2008) by the World Medical Association. All procedures were in accordance with the ethical standards of the responsible committee on human experimentation (institutional and national) and with the Helsinki Declaration of 1975 (as revised in 2008).

### Data collection in specific medical checkups

Eligible participants visited a clinic and responded to questionnaires on a marksheet regarding past history of stroke, cardiac disease, kidney disease, lifestyle habits (such as smoking, alcohol intake, and walking), and medications for hypertension, diabetes, and dyslipidemia. The questionnaire included the following 12 questions on lifestyle habits: 1) weight gain (>10 kg/20 years), 2) exercise (>30 min, twice a week, >1 year), 3) daily walking or equivalent (>1 h), 4) walking faster (than others in the same generation), 5) body weight changes (>3 kg/year), 6) eating faster (than others in the same generation), 7) eating within 2 h before going to bed (more than three times a week), 8) having a snack after dinner (more than three times a week), 9) skipping breakfast (more than three times a week), 10) daily drinking (alcohol), 11) heavy drinking (more than 60 g ethanol/day), and 12) good sleeping, by themselves. The measurements included standard medical examinations, such as height, weight, waist circumference, blood pressure, fasting blood glucose, hemoglobin A1c, triglyceride, serum high-density lipoprotein cholesterol (HDL-C), and low-density lipoprotein cholesterol (LDL-C) levels. Patients with hypertension were determined if the blood pressure was ≥140/90 mmHg or if patients were on hypotensive medication [12]. Those variables were measured in standardized manner, although they were not identical, then, collected centrally (Kanazawa Medical Association), and we referred to them. Patients with diabetes were determined if hemoglobin A1c was ≥6.5% or if the patients were on hypoglycemic medication. Stroke included ischemic and hemorrhagic stroke. We determined ASCVD as having CAD or stroke. The presence of CAD, stroke, and ASCVD was assessed based on self-reports.

### Evaluations

We assessed the factors associated with self-reported CAD, stroke, and ASCVD by adjusting the traditional risk factors, including age, sex, hypertension, diabetes, lipid-lowering therapy, body mass index (BMI), and waist circumference. In addition, we derived a lifestyle habits risk score from the 12 questions and divided the subjects into three groups based on the number of “bad habits”: 1) low group (0–3), 2) middle group (4–6), and 3) high group (7–12). We defined “bad habits” based on our results obtained by multivariable analyses (we consider “bad” if the odds ratio is greater than 1).

### Validations of self-reported outcomes

In order to validate the accuracy of self-reported outcomes, we collected objective information regarding the presence of stroke and/or CAD among a part of the participants in this study (6,674 among 47,842, 14%). We defined the presence of stroke as ischemic stroke and/or hemorrhagic stroke documented in electrical health record. Also we defined CAD as the presence of angina pectoris, myocardial infarction, or severe stenotic region(s) in the coronary artery (≥ 75% stenosis) identified either on angiography, computed tomography documented in electrical health record [13].

### Statistical analysis

Continuous variables were expressed as mean ± standard deviation, and categorical variables were expressed as number and percentage. Differences in the baseline characteristics were compared using Student’s t-test for parametric data and Mann–Whitney U-test for non-parametric data. Categorical variables were compared using the chi-square or Fisher’s exact tests. Multivariable logistic regression models were used to identify independent associations with the outcomes. Variables with p <0.10 on univariate analyses were selected for the multivariate analyses (Essentially, almost all of the variables were included in multivariable analyses). The differences between each risk score group were analyzed using a Cochran–Armitage trend test. A p value of <0.0003 [considering the number of tests (0.05 divided by 164)] was considered statistically significant, and all tests were two-tailed. All analyses were performed with R statistical software.

### Patient and public involvement

Questionnaires as well as the self-reported outcome measures in this study were determined by Japanese government. Participants of this study were not involved in the recruitment, design, or conduct of this study. The results of this study will be presented in the official website of Kanazawa Medical Association (http://www.kma.jp/cyberhospital.html).

## Results

### Validations of self-reported outcomes

Among those who had self-reported stroke, we could confirm the presence of this outcome in 442 among 6,674 individuals, and found that there was only a few discrepancies (no one among 442 individuals with documented stroke, and 2 individuals among 6,232 individuals without documented stroke). In addition to self-reported stroke, we could confirm the presence of CAD in 912 individuals, and found that there was also only a few discrepancies (1 individual among 912 individuals with documented CAD, and 2 individuals among 5,762 individuals without documented CAD).

### Characteristics of study subjects

The clinical characteristics of the study subjects are shown in **Table 1**. The subjects with self-reported CAD, stroke, or ASCVD were older and comprised a higher proportion of males, with a higher BMI, larger waist circumference, hypertension, and diabetes. On the contrary, the proportion of current smoking patients was lower in the subjects with self-reported CAD, stroke, or ASCVD, probably due to the non-smoking guidance. With respect to the lipid profile, LDL-C levels were lower in the subjects with self-reported CAD, stroke, or with ASCVD due to a high proportion of subjects on lipid-lowering therapies, although the triglyceride levels were still higher, and HDL-C levels were lower in the subjects with self-reported CAD, stroke, or ASCVD.

**Table 1:** Characteristics divided by the presence of coronary artery disease, stroke and ASCVD.

		Self-reported Coronary artery disease			Self-reported Stroke			Self-reported ASCVD		
Variable	All	Yes	No	p value	Yes	No	p value	Yes	No	p value
	(N = 47,842)	(N = 5,787)	(N = 42,055)		(N = 3,303)	(N = 44,539)		(N = 8,159)	(N = 39,683)	
Age (year)	71.4 ± 11.0	77.4 ± 9.0	70.6 ± 11.0	< 0.0003	77.0 ± 8.9	71.0 ± 11.0	< 0.0003	77.0 ± 9.0	70.3 ± 11.0	< 0.0003
Male (%)	16,913 (35.4)	2,659 (45.9)	14,254 (33.9)	< 0.0003	1,694 (51.3)	15,185 (34.1)	< 0.0003	3,848 (47.2)	13,065 (32.9)	< 0.0003
Body mass index (kg/m^2^)	22.8 ± 3.4	23.2 ± 3.5	22.7 ± 3.4	< 0.0003	23.3 ± 3.5	22.7 ± 3.4	< 0.0003	23.2 ± 3.5	22.7 ± 3.3	< 0.0003
Waist circumference (cm)	83.3 ± 9.6	84.9 ± 9.7	83.1 ± 9.6	< 0.0003	85.4 ± 9.7	83.1 ± 9.6	< 0.0003	85.0 ± 9.7	82.9 ± 9.6	< 0.0003
Triglyceride (mg/dl)	121.1 ± 75.4	124.0 ± 80.8	120.7 ± 74.6	0.002827	126.9 ± 72.7	120.6 ± 75.6	< 0.0003	124.9 ± 80.6	120.3 ± 74.8	< 0.0003
HDL cholesterol (mg/dl)	60.0 ± 15.4	55.9 ± 14.8	60.5 ± 15.4	< 0.0003	54.8 ± 14.6	60.3 ± 15.4	< 0.0003	55.7± 14.7	60.8 ± 15.4	< 0.0003
LDL cholesterol (mg/dl)	119.1 ± 30.1	110.1 ± 28.8	120.4 ± 30.1	< 0.0003	112.2 ± 28.5	119.7 ± 30.2	< 0.0003	111.3± 28.8	120.8 ± 30.1	< 0.0003
Current smoking (%)	4,600 (9.6)	428 (7.4)	4,172 (9.9)	< 0.0003	266 (8.1)	4,328 (9.7)	0.001926	638 (7.8)	3,962 (10.0)	< 0.0003
Hypertension (%)	20,968 (43.8)	3,627 (62.7)	17,341 (41.2)	< 0.0003	2,211 (66.9)	18,701 (42.0)	< 0.0003	5,193 (63.6)	15,775 (39.8)	< 0.0003
Diabetes (%)	5,578 (11.7)	1,027 (17.7)	4,551 (10.8)	< 0.0003	667 (20.2)	4,911 (11.0)	< 0.0003	1,479 (18.1)	4,099 (10.3)	< 0.0003
Lipid-lowering therapy (%)	13,405 (28.0)	1,958 (33.8)	11,447 (27.2)	< 0.0003	1,053 (31.9)	12,352 (27.7)	< 0.0003	2,716 (33.3)	10,689 (26.9)	< 0.0003
Weight gain (> 10kg per 20 years)	13,104 (27.4)	1,719 (29.7)	11,385 (27.1)	< 0.0003	1,047 (31.7)	12,057 (27.1)	< 0.0003	2,470 (30.3)	10,634 (26.8)	< 0.0003
Exercise (> 30 min, twice a week, > 1 year)	20,076 (42.0)	2,263 (39.1)	17,813 (42.4)	< 0.0003	1,221 (37.0)	18,855 (42.3)	< 0.0003	3,182 (39.0)	16,894 (42.6)	< 0.0003
Daily walking or equivalent (> 1 h)	26,774 (56.0)	3,079 (53.2)	23,695 (56.3)	< 0.0003	1,643 (49.7)	25,131 (56.4)	< 0.0003	4,317 (52.9)	22,457 (56.6)	< 0.0003
Walk faster (than the person in the same generation)	20,631 (43.1)	2,027 (35.0)	18,604 (44.2)	< 0.0003	939 (28.4)	19,692 (44.2)	< 0.0003	2,774 (34.0)	17,857 (45.0)	< 0.0003
Body weight changes (> 3kg/year)	8,969 (18.7)	1,222 (21.1)	7,747 (18.4)	< 0.0003	723 (21.9)	8,246 (18.5)	< 0.0003	1,716 (21.0)	7,253 (18.3)	< 0.0003
Eat faster (than the person in the same generation)	12,369 (25.9)	1,440 (24.9)	10,929 (26.0)	0.074	781 (23.6)	11,588 (26.0)	0.0028	1,997 (24.5)	10,372 (26.1)	0.0019
Eat dinner within 2 hours before going to bed (more than three times a week)	7,446 (15.6)	954 (16.5)	6,492 (15.4)	0.04	657 (19.9)	6,789 (15.2)	< 0.0003	1,426 (17.5)	6,020 (15.2)	< 0.0003
Have a snack after dinner (more than three times a week)	6,714 (14.0)	779 (13.5)	5,935 (14.1)	0.18	411 (12.4)	6,303 (14.2)	0.0069	1,073 (13.2)	5,641 (14.2)	0.012
Skip a breakfast more than three times a week	3,362 (7.0)	370 (6.4)	2,992 (7.1)	0.047	224 (6.8)	3,138 (7.0)	0.59	525 (6.4)	2,837 (7.1)	0.023
Daily drinking	10,679 (22.3)	1,230 (21.3)	9,449 (22.5)	0.039	722 (21.9)	9,957 (22.4)	0.52	1,767 (21.7)	8,912 (22.5)	0.11
Heavy drinking (more than 60g ethanol/day)	609 (1.3)	56 (1.0)	553 (1.3)	0.031	34 (1.0)	575 (1.3)	0.22	79 (1.0)	530 (1.3)	0.012
Good sleeping	37,950 (79.3)	4,467 (77.2)	33,123 (78.8)	< 0.0003	2,692 (81.5)	35,258 (79.2)	0.0014	6,436 (78.9)	31,514 (79.4)	0.28

Data are presented mean ± standard deviation, or number (%)

ASCVD = atherosclerotic cardiovascular diseases

With respect to the questionnaire on lifestyle habits, the subjects with self-reported CAD, stroke, or ASCVD were more likely to gain weight (>10 kg/20 years), less likely to exercise, more likely to have daily walking habits, less likely to walk faster, more likely to undergo body weight changes (>3 kg/year), less likely to eat faster, more likely to have a late dinner, having snacks after dinner, skipping breakfasts, more likely to drink daily, and more likely to drink heavily. Good sleeping habits seemed to have marginal associations.

### Factors associated with self-reported CAD, stroke, and ASCVD

We further investigated the factors associated with self-reported CAD, including the 12 questions on lifestyle habits (**Table 2**). The multivariable logistic regression analysis adjusting the traditional risk factors showed that walking faster, body weight changes, good sleeping, and lifestyle habits risk score were positively associated with the presence of self-reported CAD, whereas other factors such as exercise, daily walking, eating faster, eating dinner late, having snacks after dinner, skipping breakfast, and heavy drinking were not associated with self-reported CAD. In the analysis of subjects who experienced self-reported stroke, we observed similar but somewhat different trends compared with patients with self-reported CAD (**Table 2**). Crude OR were illustrated in **S1 Table**. With respect to self-reported stroke, factors such as walking faster, body weight changes, and lifestyle habits risk score were positively associated with self-reported stroke, whereas good sleeping was not associated with self-reported stroke. In contrast, factors such as eating faster and eating dinner late were positively associated with self-reported stroke, whereas other factors such as exercise, having snacks after dinner, skipping breakfast, and heavy drinking were not associated with self-reported stroke. Crude OR were illustrated in **S2 Table.**

**Table 2:** Factors associated with CAD, stroke, and ASCVD.

	Self-reported Coronary artery disease		Self-reported Stroke		Self-reported ASCVD	
	Adjusted OR	p value	Adjusted OR	p value	Adjusted OR	p value
	(95% CI)	(Wald’s test)	(95% CI)	(Wald’s test)	(95% CI)	(Wald’s test)
Age	1.06	<0.0003	1.04	<0.0003	1.06	<0.0003
	(1.06–1.07)		(1.04–1.05)		(1.05–1.06)	
Male	1.82	<0.0003	2.07	<0.0003	1.96	<0.0003
	(1.67–1.99)		(1.86–2.32)		(1.82–2.12)	
Hypertension	1.58	<0.0003	2.02	<0.0003	1.80	<0.0003
	(1.46–1.72)		(1.82–2.25)		(1.68–1.94)	
Diabetes	1.22	<0.0003	1.37	<0.0003	1.26	<0.0003
	(1.10–1.36)		(1.21–1.56)		(1.15–1.39)	
Dyslipidemia	1.27	<0.0003	1.11	0.073	1.23	<0.0003
	(1.16–1.38)		(0.99–1.23)		(1.14–1.33)	
Body mass index (per 1 kg/m^2^ increment)	0.99	0.553	0.96	0.004	0.98	0.022
	(0.97–1.02)		(0.93–0.99)		(0.96–1.00)	
Waist (per 1 cm increment)	1.00	0.35	1.02	0.00081	1.01	0.004
	(1.00–1.01)		(1.01–1.03)		(1.00–1.02)	
Current smoking	0.82	0.003	0.82	0.017	0.83	0.001
	(0.72–0.94)		(0.70–0.97)		(0.74–0.93)	
Weight gain (>10 kg/20 years)	1.14	0.008	1.10	0.149	1.12	0.01
	(1.04–1.26)		(0.97–1.24)		(1.03–1.22)	
Exercise (>30 min, twice a week, >1 year)	0.96	0.401	0.95	0.402	0.96	0.234
	(0.88–1.05)		(0.86–1.06)		(0.89–1.03)	
Daily walking or equivalent (>1 h)	1.07	0.141	0.87	0.008	1.00	0.929
	(0.98–1.16)		(0.78–0.96)		(0.93–1.08)	
Walking faster (than people of the same generation)	0.83	<0.0003	0.61	<0.0003	0.74	<0.0003
	(0.76–0.9)		(0.55–0.68)		(0.69–0.79)	
Body weight changes (>3 kg/year)	1.25	<0.0003	1.35	<0.0003	1.26	<0.0003
	(1.14–1.38)		(1.20–1.52)		(1.16–1.37)	
Eating faster (than people of the same generation)	1.00	0.922	1.20	<0.0003	1.09	0.003
	(0.94–1.07)		(1.11–1.30)		(1.03–1.15)	
Eating dinner within 2 h before going to bed (more than three times a week)	1.03	0.622	1.31	<0.0003	1.14	0.003
	(0.93–1.13)		(1.16–1.48)		(1.05–1.25)	
Having a snack after dinner (more than three times a week)	1.07	0.272	0.91	0.217	1.01	0.884
	(0.95–1.20)		(0.78–1.06)		(0.91–1.12)	
Skipping breakfast more than three times a week	1.06	0.447	0.99	0.928	1.03	0.68
	(0.91–1.24)		(0.82–1.20)		(0.90–1.18)	
Daily drinking	0.87	0.002	0.76	<0.0003	0.83	<0.0003
	(0.80–0.95)		(0.68–0.85)		(0.76–0.89)	
Heavy drinking (more than 60 g ethanol/day)	0.97	0.825	0.9	0.579	0.90	0.417
	(0.72–1.30)		(0.62–1.31)		(0.70–1.16)	
Good sleeping	0.81	<0.0003	1.00	0.956	0.86	<0.0003
	(0.74–0.89)		(0.88–1.12)		(0.79–0.93)	
Lifestyle habits risk score	1.10	<0.0003	1.18	<0.0003	1.12	<0.0003
	(1.08–1.11)		(1.15–1.20)		(1.10–1.13)	

ORs were calculated after adjusting for age, gender, hypertension, diabetes, lipid-lowering therapy, BMI, and waist circumference.

ASCVD = atherosclerotic cardiovascular diseases, CI = confidence interval, OR = odds ratio

Finally, we investigated the factors associated with self-reported ASCVD (**Table 2**) and found that walking faster, body weight changes, daily drinking, good sleeping, and lifestyle habits risk score were positively associated with self-reported ASCVD, while other factors such as exercise, daily walking, having snacks after dinner, skipping breakfast, and heavy drinking were not associated with self-reported ASCVD. Crude OR were illustrated in **S3 Table.**

### Lifestyle habits risk score

**Fig 2:**
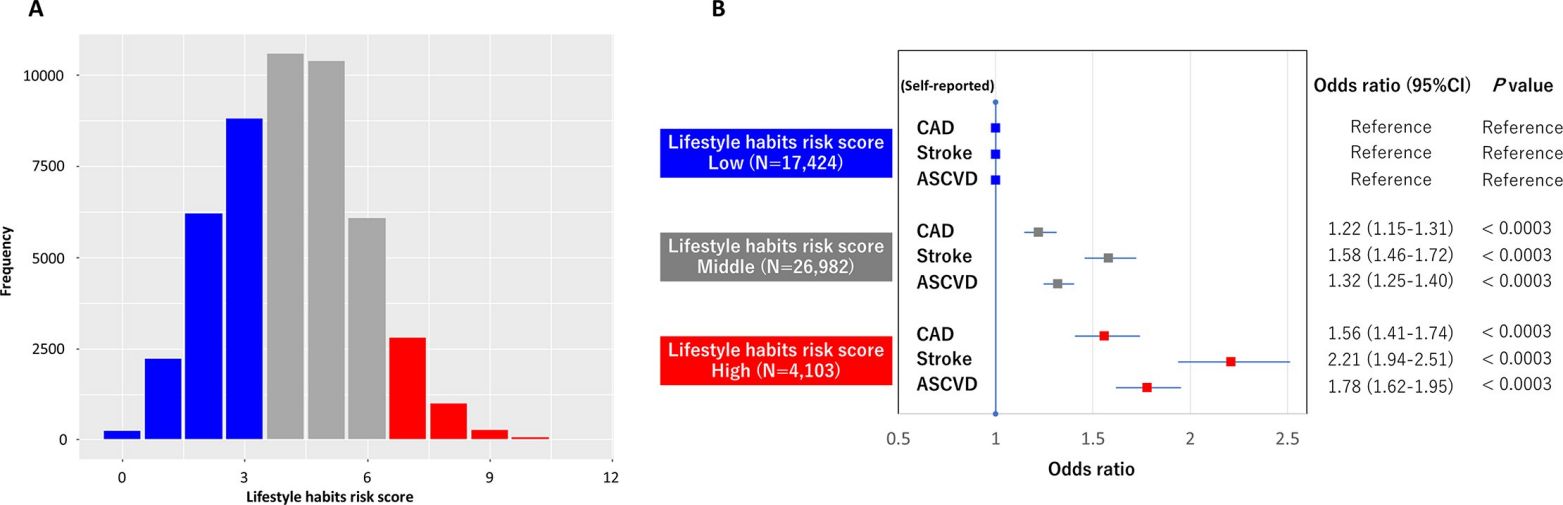
Impact of lifestyle habits risk score on the prevalence of self-reported CAD, stroke, and ASCVD. **A. Distribution of Lifestyle Habits Risk Score among the Japanese General Population.** The X-axis represents lifestyle habits risk scores comprising 12 questions related to lifestyle habits (number of “bad habits”). The Y-axis represents frequency. Blue indicates the Low group (lifestyle habits risk score 0 to 3). Gray indicates the Middle group (lifestyle habits risk score 4 to 6). Red indicates the High group (lifestyle habits risk score 7–12). **B. Association between lifestyle habits risk score and the prevalence of self-reported CAD, stroke, and ASCVD.** Odds ratios for each outcome were calculated using logistic regression models adjusted for traditional risk factors, including age, gender, hypertension, diabetes, lipid-lowering therapy, body mass index, and waist circumference. CAD = coronary artery disease; ASCVD = atherosclerotic cardiovascular diseases.

The distribution of the lifestyle habits risk score is illustrated in **Fig 2A**. The odds for self-reported CAD were significantly higher in the middle and high groups (adjusted OR = 1.22, 95% CI = 1.15–1.31, p < 0.0003, adjusted OR = 1.56, 95% CI = 1.41–1.74, p < 0.0003, respectively, p-trend < 0.001, **Fig 2B**) than that in the low group. Similarly, the odds for self-reported stroke in the middle and high groups were significantly higher than that in the low group (adjusted OR = 1.58, 95% CI = 1.46–1.72, p < 0.0003, adjusted OR = 2.21, 95% CI = 1.94–2.51, p < 0.0003, respectively, p-trend < 0.001, **Fig 2B**). Finally, similar trends were observed for self-reported ASCVD outcomes, where the odds for ASCVD were significantly higher in the middle and high groups than that in the low group (adjusted OR = 1.32, 95% CI = 1.25–1.40, p < 0.0003, adjusted OR = 1.78, 95% CI = 1.62–1.95, p < 0.0003, respectively, p-trend < 0.001, **Fig 2B**).

### Difference between each gender

We additionally assessed the effect of gender on the results. We divided the subjects into two groups based on gender and investigated the association between questionnaires and the presence of self-reported ASCVD. We found no significant between-group difference in this respect (**S4 Table, S5 Table**). In addition, there was no essential difference between men and women with respect to the impact of lifestyle habits risk scores on the prevalence of self-reported ASCVD.

## Discussion

Using a large dataset from the community-based specific medical checkups, we observed the following: 1) most of the questions on lifestyle habits were independently associated with the presence of self-reported ASCVD and 2) lifestyle habits risk scores, comprising 12 questions on lifestyle habits, were independently associated with the presence of self-reported ASCVD.

There are several reports investigating the association between the lifestyle habits and cardiovascular events in the Japanese population [14, 15]. However, there is little data investigating the association between the questions employed in nation-wide community-based medical checkups and the presence of ASCVD.

It should be noted that lifestyle habits that have shown to be associated with ASCVD in the current study can be evaluated without any invasion or cost. Accordingly, attention should be paid to such lifestyle habits that generally could be changed, although in this cross-sectional study, we could not assess the causality of the questionnaires. Nevertheless, our results suggest that any intervention that has been shown to reduce the risk (such as lipid-lowering therapy, treatment of hypertension, or smoking cessation) should be considered if the score is high. In addition, our results suggested that the individuals with more bad lifestyle habits are more likely to exhibit ASCVD. Accordingly, we could consider assessments for the presence of ASCVD, including angiogram for such “high-risk” individuals.

Interestingly, several lifestyle habits that are believed to be good or bad, such as exercise, having a snack after dinner, or skipping a breakfast more than three times a week, had no association with self-reported ASCVD outcomes in this study. On the other hand, walking faster, body weight changes within a year, and daily drinking were strongly associated with self-reported ASCVD outcomes. Regarding the association between exercise and self-reported ASCVD, the proportion of individuals with a habit of exercise was significantly smaller among patients with self-reported ASCVD. However, this association disappeared after adjusting for confounding factors. Accordingly, we assume that potential confounding factors such as age and gender may affect the association between them. On the contrary, the questionnaire item “walking faster than people of the same generation” accounts for the effect of age and shows a true association.

In this study, we have validated the accuracy of self-reported ASCVD using a subgroup whose status had been assessed objectively. In Japan, we have free compulsory education system where every Japanese children receive the same education for 9 years. Accordingly, the term stroke and coronary artery disease (and its combination) should be identified quite uniformly in this country.

This study has several limitations. First, this study investigated a part of population living in Kanazawa city and not a nationwide dataset, which could potentially affect the results. In addition, our cohort mainly consisted of females, which may have also potentially affected the results. Actually, this health checkups were offered only for individuals without “regular” occupation. In Japan, “regular” worker must undergo health checkups offered by their workplaces, instead of this specific health checkups. Actually, more male are working regularly than female in Japan. However, we believe that our results using large sample size could represent Japanese population. Second, the responses to the questionnaire are subjective compared with the objective laboratory data. It is quite difficult to objectively and quantitatively measure “lifestyle.” Third, CAD and stroke were determined using medical questionnaires, which could lead to underestimating the presence of ASCVD. However, other established risk factors, such as age, sex, and lipids, were robustly associated with the “self-reported” ASCVD, suggesting that these assessments were acceptable. Fourth, due to the cross-sectional study design, we could not assess the causality. For example, we observed the negative association between the presence of ASCVD and current smoking, probably due to the smoking cessation after the events. For the smoking habit, we only considered “current” smokers in this study. In other words, many former smokers who quit smoking after the occurrence of ASCVD are classified as non-current smokers. In addition, the smokers may not survive their CAD, stroke or ASCVD to the same degree as non-smokers, which should lead to our paradoxical observation. We believe that those are the main reasons for our paradoxical results. Fifth, we could not assess the pattern of diet, which would be interesting to see. Sixth, the questionnaires used in this study do not include the issues relating with diet, which should be significantly associated with the development of ASCVD. Sixth, we have any missing data among 666/48,508 (1.3%) individuals, which could affect our results. However, only a portion of individuals who failed to answer those questionnaires (143 among 666 individuals, and other variables, such as serum cholesterol, and or blood pressure are missing among 544 individuals, the reason of which is unknown. Seventh, the technique used in measurements for variables were not identical in this study. However, all of the measurements for biochemical parameters were performed by standardized enzymatic methods. Thus, we believe that error should not be large. Finally, although we assessed the association between questions on lifestyle habits and self-reported ASCVD, the causalities remain unknown. Further intervention trials are needed to validate these causalities.

## Conclusions

In conclusion, questionnaires on lifestyle habits were associated with self-reported ASCVD.

## Supporting information

S1 Table Factors associated with CAD. (DOC)

S2 Table Factors associated with stroke. (DOC)

S3 Table Factors associated with ASCVD. (DOC)

S4 Table Factors associated with self-reported ASCVD disaggregated by gender. (DOC)

S5 Table Impact of lifestyle habits risk score on self-reported ASCVD disaggregated by gender. (DOC)
