# Supplementary material for: Associations between questionnaires on lifestyle and atherosclerotic cardiovascular disease in a Japanese general population: A cross-sectional study
Source: PLoS One. 2018 Nov 28;13(11):e0208135. doi: 10.1371/journal.pone.0208135 (PMC6261639; doi:10.1371/journal.pone.0208135)
Supplement: S1 Table — (DOC) [file pone.0208135.s001.doc]

**S1 Table.**

|  | crude OR (95% CI) | adjusted OR (95% CI) | P value (Wald's test) |
| --- | --- | --- | --- |
| Age | 1.07 (1.06–1.07) | 1.06 (1.06–1.07) | <2 × 10^−16^ |
| Male | 1.89 (1.75–2.03) | 1.82 (1.67–1.99) | <2×10^−16^ |
| Hypertension | 2.42 (2.24–2.61) | 1.58 (1.46–1.72) | <2×10^−16^ |
| Diabetes | 1.79 (1.62–1.98) | 1.22 (1.10–1.36) | 0.000228 |
| Dyslipidemia | 1.51 (1.40–1.64) | 1.27 (1.16–1.38) | 5.8 × 10^−8^ |
| Body mass index (per 1kg/m^2^ increment) | 1.04 (1.04–1.06) | 0.99 (0.97–1.02) | 0.553 |
| Waist (per 1cm increment) | 1.02 (1.02–1.03) | 1.00 (1.00–1.01) | 0.35 |
| Current Smoking | 0.74 (0.66–0.84) | 0.82 (0.72–0.94) | 0.003 |
| Weight gain (>10 kg/20 years) | 1.27 (1.17–1.37) | 1.14 (1.04–1.26) | 0.008 |
| Exercise (>30 min, twice a week, >1 year) | 0.97 (0.90–1.05) | 0.96 (0.88–1.05) | 0.401 |
| Daily walking or equivalent (>1 h) | 0.99 (0.92–1.07) | 1.07 (0.98–1.16) | 0.141 |
| Walk faster (than the person in the same generation) | 0.73 (0.68–0.79) | 0.83 (0.76–0.9) | 7.1 × 10^−6^ |
| Body weight changes (>3 kg/year) | 1.17 (1.07–1.28) | 1.25 (1.14–1.38) | 3.8 × 10^−6^ |
| Eat faster than the person in the same generation | 1.15 (1.09–1.22) | 1.00 (0.94–1.07) | 0.922 |
| Eat dinner within 2 h before going to bed (more than three times a week) | 1.06 (0.96–1.16) | 1.03 (0.93–1.13) | 0.622 |
| Have a snack after dinner (more than three times a week) | 0.91 (0.81–1.01) | 1.07 (0.95–1.20) | 0.272 |
| Skip a breakfast more than three times a week | 0.85 (0.74–0.99) | 1.06 (0.91–1.24) | 0.447 |
| Daily drinking | 1.03 (0.95–1.11) | 0.87 (0.80–0.95) | 0.002 |
| Heavy drinking (more than 60 g ethanol/day) | 0.78 (0.59–1.04) | 0.97 (0.72–1.30) | 0.825 |
| Good sleeping | 0.93 (0.85–1.02) | 0.81 (0.74–0.89) | 1.2 × 10^−5^ |
| Lifestyle habits risk score | 1.08  (1.06–1.09) | 1.10  (1.08–1.11) | <2 × 10^−16^ |

OR = odds ratio.
