# Supplementary material for: Associations between questionnaires on lifestyle and atherosclerotic cardiovascular disease in a Japanese general population: A cross-sectional study
Source: PLoS One. 2018 Nov 28;13(11):e0208135. doi: 10.1371/journal.pone.0208135 (PMC6261639; doi:10.1371/journal.pone.0208135)
Supplement: S2 Table — (DOC) [file pone.0208135.s002.doc]

**S2 Table.**

|  | crude OR (95% CI) | Adjusted OR  (95% CI) | P value (Wald's test) |
| --- | --- | --- | --- |
| Age | 1.06 (1.06–1.07) | 1.04  (1.04–1.05) | <2 × 10^−16^ |
| Male | 2.12 (1.92–2.33) | 2.07 (1.86–2.32) | <2 × 10^−16^ |
| Hypertension | 2.89 (2.62–3.18) | 2.02 (1.82–2.25) | <2 × 10^−16^ |
| Diabetes | 2.03 (1.80–2.29) | 1.37 (1.21–1.56) | 1.2 × 10^−6^ |
| Dyslipidemia | 1.33 (1.20–1.48) | 1.11 (0.99–1.23) | 0.07300 |
| Body mass index (per 1kg/m^2^ increment) | 1.05 (1.03–1.06) | 0.96 (0.93–0.99) | 0.00400 |
| Waist (per 1cm increment) | 1.03 (1.02–1.03) | 1.02 (1.01–1.03) | 0.00081 |
| Current Smoking | 0.82 (0.71–0.96) | 0.82 (0.70–0.97) | 0.01700 |
| Weight gain (>10 kg per 20 years) | 1.28 (1.16–1.41) | 1.10 (0.97–1.24) | 0.14900 |
| Exercise (>30 min, twice a week, >1 year) | 0.83 (0.76–0.91) | 0.95 (0.86–1.06) | 0.40200 |
| Daily walking or equivalent (>1 h) | 0.77 (0.70–0.84) | 0.87 (0.78–0.96) | 0.00800 |
| Walk faster (than the person in the same generation) | 0.51 (0.46–0.56) | 0.61 (0.55–0.68) | <2 × 10^−16^ |
| Body weight changes (>3 kg/year) | 1.31 (1.17–1.46) | 1.35 (1.20–1.52) | 4.3 × 10^−7^ |
| Eat faster than the person in the same generation | 1.38 (1.28–1.48) | 1.20 (1.11–1.30) | 7.5 × 10^−6^ |
| Eat dinner within 2 h before going to bed (more than three times a week) | 1.34 (1.20–1.50) | 1.31 (1.16–1.48) | 8.7 × 10^−6^ |
| Have a snack after dinner (more than three times a week) | 0.80 (0.69–0.93) | 0.91 (0.78–1.06) | 0.21700 |
| Skip a breakfast more than three times a week | 0.87 (0.72–1.04) | 0.99 (0.82–1.20) | 0.92800 |
| Daily drinking | 1.02 (0.92–1.12) | 0.76 (0.68–0.85) | 2.1 × 10^−6^ |
| Heavy drinking (more than 60 g ethanol/day) | 0.79 (0.55–1.14) | 0.90 (0.62–1.31) | 0.57900 |
| Good sleeping | 1.06 (0.95–1.20) | 1.00 (0.88–1.12) | 0.95600 |
| Lifestyle habits risk score | 1.14  (1.12–1.17) | 1.18  (1.15–1.20) | <2 × 10^−16^ |

OR = odds ratio.
