# Supplementary material for: Associations between questionnaires on lifestyle and atherosclerotic cardiovascular disease in a Japanese general population: A cross-sectional study
Source: PLoS One. 2018 Nov 28;13(11):e0208135. doi: 10.1371/journal.pone.0208135 (PMC6261639; doi:10.1371/journal.pone.0208135)
Supplement: S3 Table — (DOC) [file pone.0208135.s003.doc]

**S3 Table.**

|  | Crude OR (95%CI) | Adjusted OR (95%CI) | P value (Wald's test) |
| --- | --- | --- | --- |
| Age | 1.07 (1.06–1.07) | 1.06 (1.05–1.06) | <2 × 10^−16^ |
| Male | 2.01 (1.88–2.14) | 1.96 (1.82–2.12) | <2 × 10^−16^ |
| Hypertension | 2.68 (2.51–2.87) | 1.80 (1.68–1.94) | <2 × 10^−16^ |
| Diabetes | 1.89 (1.73–2.06) | 1.26 (1.15–1.39) | 1.2 × 10^−6^ |
| Dyslipidemia | 1.48 (1.38–1.59) | 1.23 (1.14–1.33) | 6.8 × 10^−8^ |
| Body mass index (per 1kg/m^2^ increment) | 1.05 (1.04–1.06) | 0.98 (0.96–-1.00) | 0.022 |
| Waist (per 1cm increment) | 1.03 (1.02–1.03) | 1.01 (1.00–1.02) | 0.004 |
| Current smoking | 0.78 (0.71–0.87) | 0.83 (0.74–-0.93) | 0.001 |
| Weight gain (>10 kg per 20 years) | 1.27 (1.19-1.36) | 1.12 (1.03-1.22) | 0.01 |
| Exercise (>30 min, twice a week, >1 year) | 0.93 (0.87–0.99) | 0.96 (0.89–1.03) | 0.234 |
| Daily walking or equivalent (>1 h) | 0.92 (0.87–0.98) | 1.00 (0.93–1.08) | 0.929 |
| Walk faster (than the person in the same generation) | 0.65 (0.61–0.69) | 0.74 (0.69–0.79) | <2 × 10^−16^ |
| Body weight changes (>3 kg/year) | 1.18 (1.09–1.28) | 1.26 (1.16–1.37) | 5.3 × 10^−8^ |
| Eat faster than the person in the same generation | 1.24 (1.18–1.31) | 1.09 (1.03–1.15) | 0.003 |
| Eat dinner within 2 h before going to bed (more than three times a week) | 1.17 (1.08–1.26) | 1.14 (1.05–1.25) | 0.003 |
| Have a snack after dinner (more than three times a week) | 0.85 (0.78–0.94) | 1.01  (0.91–1.12) | 0.884 |
| Skip a breakfast more than three times a week | 0.84 (0.74–0.95) | 1.03 (0.90–1.18) | 0.68 |
| Daily drinking | 1.04 (0.97–1.11) | 0.83 (0.76–0.89) | 1.5 × 10^−6^ |
| Heavy drinking (more than 60 g ethanol/day) | 0.76 (0.60–0.97) | 0.90 (0.70–1.16) | 0.417 |
| Good sleeping | 0.97 (0.90–1.05) | 0.86 (0.79–0.93) | 0.000212 |
| Lifestyle habits risk score | 1.09  (1.07–1.10) | 1.12  (1.10–1.13) | <2×10^−16^ |

OR = odds ratio.
