# Supplementary material for: Associations between questionnaires on lifestyle and atherosclerotic cardiovascular disease in a Japanese general population: A cross-sectional study
Source: PLoS One. 2018 Nov 28;13(11):e0208135. doi: 10.1371/journal.pone.0208135 (PMC6261639; doi:10.1371/journal.pone.0208135)
Supplement: S4 Table — (DOC) [file pone.0208135.s004.doc]

**S4 Table.**

|  | Male | |  | Female | |
| --- | --- | --- | --- | --- | --- |
|  | Adjusted OR (95% CI) | pvalue (Wald’s test) |  | Adjusted OR (95% CI) | p value (Wald’s test) |
| Weight gain (>10 kg/20 years) | 1.06 (0.97–1.17) | 0.2018 |  | 1.18 (1.08–1.30) | 0.000381 |
| Exercise (>30 min, twice a week, >1 year) | 1.06 (0.97–1.15) | 0.19521 |  | 1.05 (0.98–1.14) | 0.178432 |
| Daily walking or equivalent (>1 h) | 0.97 (0.89–1.06) | 0.53216 |  | 0.97 (0.90–1.05) | 0.469655 |
| Walking faster (than people of the same generation) | 1.51 (1.39–1.63) | <0.0002 |  | 1.31 (1.21–1.41) | <0.0003 |
| Body weight changes (>3 kg/year) | 1.29 (1.17–1.41) | <0.0002 |  | 1.22 (1.11–1.33) | <0.0003 |
| Eating faster (than people of the same generation) | 0.93 (0.85–1.01) | 0.08619 |  | 0.91 (0.84–0.99) | 0.027137 |
| Eating dinner within 2 h before going to bed (more than three times a week) | 1.14 (1.04–1.25) | 0.00601 |  | 1.15 (1.04–1.27) | 0.006151 |
| Having a snack after dinner (more than three times a week) | 1.00 (0.89–1.12) | 0.97775 |  | 1.07 (0.97–1.18) | 0.177989 |
| Skipping breakfast more than three times a week | 1.06 (0.91–1.23) | 0.43777 |  | 1.01 (0.87–1.17) | 0.905582 |
| Daily drinking | 1.23 (1.13–1.32) | <0.0003 |  | 1.02 (0.90–1.17) | 0.718611 |
| Heavy drinking (more than 60 g ethanol/day) | 0.87 (0.66–1.14) | 0.32642 |  | 1.10 (0.58–1.92) | 0.74117 |
| Good sleeping | 1.08 (0.98–1.19) | 0.09886 |  | 1.13 (1.03–1.22) | 0.004596 |
| Lifestyle habits risk score | 1.11 (1.09–1.13) | <0.0003 |  | 1.09 (1.07–1.11) | <0.0003 |

OR = odds ratio, CI = confidence interval.

ORs were calculated after adjusting for traditional risk factors, including age, hypertension, diabetes, lipid-lowering therapy, body mass index, and waist circumference.
