# Supplementary material for: Associations between questionnaires on lifestyle and atherosclerotic cardiovascular disease in a Japanese general population: A cross-sectional study
Source: PLoS One. 2018 Nov 28;13(11):e0208135. doi: 10.1371/journal.pone.0208135 (PMC6261639; doi:10.1371/journal.pone.0208135)
Supplement: S5 Table — (DOC) [file pone.0208135.s005.doc]

**S5 Table.**

| Outcome | Male | | |  | Female | | |
| --- | --- | --- | --- | --- | --- | --- | --- |
|  | Lifestyle habits risk score | | |  | Lifestyle habits risk score | | |
|  | Low (N = 7,223) | Middle (N = 8,425) | High (N = 1,265) |  | Low (N = 10,201) | Middle (N = 18,557) | High (N = 2,838) |
| ASCVD | Reference | 1.31 (1.21–1.42) | 1.61 (1.40–1.86) |  | Reference | 1.24 (1.15–1.34) | 1.60 (1.40–1.81) |

ASCVD = atherosclerotic cardiovascular diseases.
